# Dihydro­myricetin hexa­acetate

**DOI:** 10.1107/S1600536810037578

**Published:** 2010-09-25

**Authors:** Wei Li, Mohan Bhadbhade, James Hook, Jian Zhao

**Affiliations:** a College of Biological Engineering, Hubei University of Technology, Wuhan 430068, People’s Republic of China; b Mark Wainwright Analytical Centre, The University of New South Wales, Sydney, NSW 2052, Australia; c School of Chemical Engineering, The University of New South Wales, Sydney, NSW 2052, Australia

## Abstract

In the title compound, C_27_H_24_O_14_, also known as 2,3-di­acetoxy-5-[(2*RS*,3*RS*)-3,5,7-triacetoxy-4-oxochromen-2-yl]phenyl acetate, the heterocyclic ring adopts a distorted half-chair conformation, with two C atoms displaced by 0.1775 (16) and −0.5950 (16) Å from the mean plane of the other four atoms. The dihedral angle between the aromatic rings is 57.81 (8)°. In the crystal, the mol­ecules inter­act by C—H⋯O bonds, aromatic π–π stacking [centroid–centroid separation = 3.6206 (9) Å] and C—H⋯π inter­actions.

## Related literature

For the crystal structure of dihydro­myricetin, see: Xu *et al.* (2007 ▶). For the properties of dihydro­myricetin, see: Li *et al.* (2006 ▶); Liu *et al.* (2009 ▶), Gao *et al.* (2009 ▶).
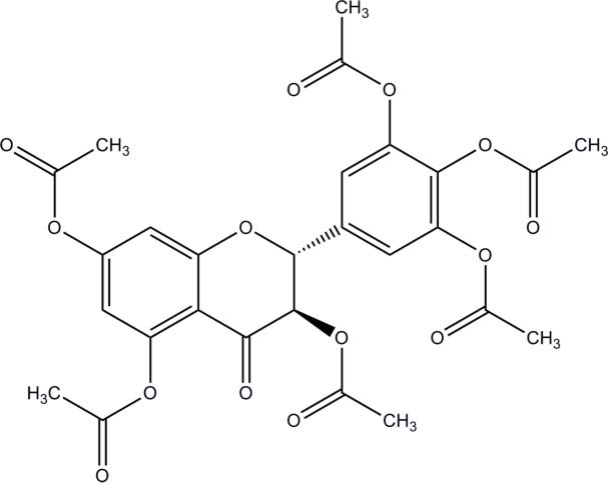


## Experimental

### 

#### Crystal data

C_27_H_24_O_14_
*M* *_r_* = 572.46
Triclinic, 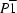

*a* = 7.7979 (2) Å
*b* = 11.6652 (3) Å
*c* = 16.2083 (4) Å
α = 96.889 (1)°
β = 97.600 (1)°
γ = 109.085 (1)°
*V* = 1359.97 (6) Å^3^
*Z* = 2
Mo *K*α radiation
μ = 0.12 mm^−1^
*T* = 150 K
0.21 × 0.21 × 0.09 mm

#### Data collection

Bruker Kappa APEXII CCD diffractometer
Absorption correction: multi-scan (*SADABS*; Bruker, 2001 ▶) *T*_min_ = 0.976, *T*_max_ = 0.989
16120 measured reflections
5856 independent reflections
4761 reflections with *I* > 2σ(*I*)
*R*_int_ = 0.041

#### Refinement

*R*[*F* ^2^ > 2σ(*F* ^2^)] = 0.040
*wR*(*F* ^2^) = 0.110
*S* = 1.03
5856 reflections
377 parameters
H-atom parameters constrained
Δρ_max_ = 0.30 e Å^−3^
Δρ_min_ = −0.32 e Å^−3^

### 

Data collection: *APEX2* (Bruker, 2007 ▶); cell refinement: *SAINT* (Bruker, 2007 ▶); data reduction: *SAINT*; program(s) used to solve structure: *SHELXS97* (Sheldrick, 2008 ▶); program(s) used to refine structure: *SHELXL97* (Sheldrick, 2008 ▶); molecular graphics: *SHELXTL-Plus* (Sheldrick, 2008 ▶); software used to prepare material for publication: *SHELXTL-Plus*.

## Supplementary Material

Crystal structure: contains datablocks I, global. DOI: 10.1107/S1600536810037578/hb5603sup1.cif

Structure factors: contains datablocks I. DOI: 10.1107/S1600536810037578/hb5603Isup2.hkl

Additional supplementary materials:  crystallographic information; 3D view; checkCIF report

## Figures and Tables

**Table 1:** Hydrogen-bond geometry (Å, °) *Cg*3 is the centroid of the C16–C21 ring.

*D*—H⋯*A*	*D*—H	H⋯*A*	*D*⋯*A*	*D*—H⋯*A*
C1—H1⋯O6^i^	0.95	2.53	3.2847 (19)	136
C8—H8⋯O3^i^	1.00	2.33	3.2833 (18)	158
C11—H11*A*⋯O2^ii^	0.98	2.40	3.347 (2)	161
C13—H13*A*⋯O4^iii^	0.98	2.44	3.417 (2)	176
C15—H15*B*⋯O4^iv^	0.98	2.59	3.232 (2)	124
C23—H23*B*⋯*Cg*3^v^	0.98	2.86	3.7598 (18)	153

Symmetry codes: (i) 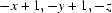; (ii) 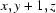; (iii) 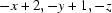; (iv) 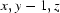; (v) 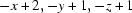.

**Fig. 1.**
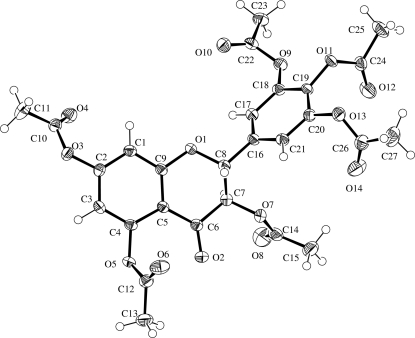
View of the (R,R) enantiomer of (I). The displacement ellipsoids shown at 50% probability level.

**Fig. 2.**
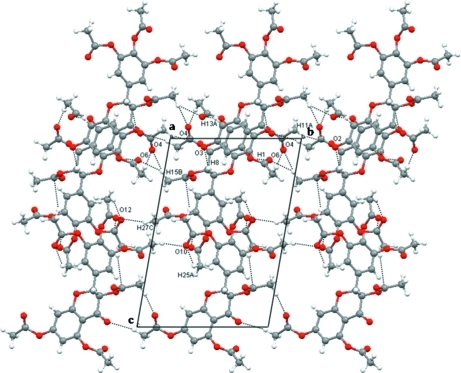
Packing of the molecules viewed down the a axis showing the C—H···O interactions.

## supplementary crystallographic information

### Comment

Dihydromyricetin is the principal flavonoid of *Ampelopsis grossedentata*, a vine which grows abundantly in southern China. The compound is a strong anti-oxidant with many reported health-promoting properties and, therefore, is emerging as a promising functional ingredient for use in a number of pharmaceutical and food applications (Li *et al.*, 2006). However, the compound is poorly soluble in both water and fat, which limits its applications (Gao *et al.*, 2009). Acetylation could be one way how to improve its lipid solubility. Although the single-crystal structure of dihydromyricetin itself is known (Xu *et al.*, 2007), none of its derivatives have to date been structurally characterized. Herein we report the structure of the hexaacetate of dihydromyricetin (Fig. 1).

Though the title structure is of the biological origin, it crystallizes in a structure where both enantiomers are present. Fig. 1 shows the view of the (*R*, *R*) enantiomer. The torsion angle between the benzopyrone and the phenyl ring (O1—C8—C16—C17) is -36.2 (2)°. The crystal packing (Tab. 1, Fig. 2) contains a network of C—H···O interactions. Moreover, there are also present π-electron ring - π-electron ring interactions between the adjacent rings C1//C2//C3//C4//C5//C9 [symmetry code: 1-*x*,1-*y*, -*z*] as indicates the distance between the centroids that equals to 3.6206 (9)Å.

### Experimental

The crystals of dihydromyricetin (1 g), prepared as described by Xu *et al.* (2007), were added by parts to a mixture of acetic anhydride (6 ml) and pyridine (1 ml) maintained at 75°C in the water bath. Each addition was done after the crystals of dihydromyricetin that had been added previously completely dissolved. The mixture was stirred for 30 min and upon addition of chilled water (120 ml), yielded an oily precipitate, which solidified in 15 min. After decanting the supernatant and washing the precipitate with water, the precipitate was collected and dried at 55°C for 24 h to afford a light yellow solid. A portion (50 mg) was dissolved in warm methanol, and yielded, on standing for several days at ambient temperature, colourless plates of (I) (m.p. 436 - 440 K).

### Refinement

All the H atoms were located in the difference electron density map. However, the H atoms were placed into the idealized positions with d(C—H) = 0.95 Å for the aryl, 0.980 Å for the methyl and 1.000 Å for the methine hydrogens. The *U*_iso_(H) values were constrained to be 1.2*U*_eq_ for the aryl and methine H atoms while 1.5*U*_eq_ for the methyl H atoms.

### Crystal data

**Table:**

C_27_H_24_O_14_	*Z* = 2
*M**_r_* = 572.46	*F*(000) = 596
Triclinic, *P*1	*D*_x_ = 1.398 Mg m^−^^3^
Hall symbol: -P 1	Melting point = 436–440 K
*a* = 7.7979 (2) Å	Mo *K*α radiation, λ = 0.71073 Å
*b* = 11.6652 (3) Å	Cell parameters from 7407 reflections
*c* = 16.2083 (4) Å	θ = 2.5–30.4°
α = 96.889 (1)°	µ = 0.12 mm^−^^1^
β = 97.600 (1)°	*T* = 150 K
γ = 109.085 (1)°	Plate, colourless
*V* = 1359.97 (6) Å^3^	0.21 × 0.21 × 0.09 mm

### Data collection

**Table:**

Bruker Kappa APEXII CCD diffractometer	5856 independent reflections
Radiation source: fine-focus sealed tube	4761 reflections with *I* > 2σ(*I*)
graphite	*R*_int_ = 0.041
φ scans and ω scans with κ offsets	θ_max_ = 27.0°, θ_min_ = 2.9°
Absorption correction: multi-scan (*SADABS*; Bruker, 2001)	*h* = −9→9
*T*_min_ = 0.976, *T*_max_ = 0.989	*k* = −14→14
16120 measured reflections	*l* = −20→20

### Refinement

**Table:**

Refinement on *F*^2^	Secondary atom site location: difference Fourier map
Least-squares matrix: full	Hydrogen site location: difference Fourier map
*R*[*F*^2^ > 2σ(*F*^2^)] = 0.040	H-atom parameters constrained
*wR*(*F*^2^) = 0.110	*w* = 1/[σ^2^(*F*_o_^2^) + (0.0452*P*)^2^ + 0.5506*P*] where *P* = (*F*_o_^2^ + 2*F*_c_^2^)/3
*S* = 1.03	(Δ/σ)_max_ = 0.001
5856 reflections	Δρ_max_ = 0.30 e Å^−^^3^
377 parameters	Δρ_min_ = −0.32 e Å^−^^3^
0 restraints	Extinction correction: *SHELXL*, Fc^*^=kFc[1+0.001xFc^2^λ^3^/sin(2θ)]^-1/4^
90 constraints	Extinction coefficient: 0.0070 (16)
Primary atom site location: structure-invariant direct methods	

### Special details

**Table:**

Geometry. All e.s.d.'s (except the e.s.d. in the dihedral angle between two l.s. planes) are estimated using the full covariance matrix. The cell e.s.d.'s are taken into account individually in the estimation of e.s.d.'s in distances, angles and torsion angles; correlations between e.s.d.'s in cell parameters are only used when they are defined by crystal symmetry. An approximate (isotropic) treatment of cell e.s.d.'s is used for estimating e.s.d.'s involving l.s. planes.
Refinement. Refinement of *F*^2^ against ALL reflections. The weighted *R*-factor *wR* and goodness of fit *S* are based on *F*^2^, conventional *R*-factors *R* are based on *F*, with *F* set to zero for negative *F*^2^. The threshold expression of *F*^2^ > σ (*F*^2^) is used only for calculating *R*-factors (gt) *etc*. and is not relevant to the choice of reflections for refinement. *R*-factors based on *F*^2^ are statistically about twice as large as those based on *F*, and *R*- factors based on ALL data will be even larger.

### Fractional atomic coordinates and isotropic or equivalent isotropic displacement parameters (Å^2^)

**Table:**

	*x*	*y*	*z*	*U*_iso_*/*U*_eq_	
O1	0.72782 (15)	0.50439 (9)	0.17597 (6)	0.0248 (2)	
O2	0.82930 (16)	0.23522 (10)	0.02789 (7)	0.0285 (3)	
O3	0.69164 (15)	0.73102 (9)	−0.04552 (6)	0.0249 (2)	
O4	0.88884 (16)	0.87775 (10)	0.06016 (7)	0.0332 (3)	
O5	0.81318 (15)	0.36393 (9)	−0.10513 (6)	0.0243 (2)	
O6	0.53693 (17)	0.21507 (11)	−0.11517 (8)	0.0395 (3)	
O7	0.77402 (16)	0.20741 (10)	0.18599 (7)	0.0276 (3)	
O8	1.0734 (2)	0.22913 (14)	0.20952 (9)	0.0503 (4)	
O9	0.86595 (16)	0.57686 (10)	0.49672 (6)	0.0275 (3)	
O10	0.84901 (17)	0.73527 (11)	0.43165 (7)	0.0320 (3)	
O11	0.57423 (16)	0.40577 (11)	0.53218 (6)	0.0275 (3)	
O12	0.7581 (2)	0.30683 (15)	0.57861 (8)	0.0540 (4)	
O13	0.36896 (15)	0.19986 (10)	0.41469 (7)	0.0294 (3)	
O14	0.50639 (19)	0.06354 (12)	0.37879 (10)	0.0475 (4)	
C1	0.7183 (2)	0.61969 (13)	0.06911 (9)	0.0220 (3)	
H1	0.6996	0.6799	0.1083	0.026*	
C2	0.7256 (2)	0.63424 (13)	−0.01355 (9)	0.0206 (3)	
C3	0.7503 (2)	0.54634 (13)	−0.07242 (9)	0.0213 (3)	
H3	0.7523	0.5575	−0.1294	0.026*	
C4	0.77163 (19)	0.44346 (13)	−0.04645 (9)	0.0200 (3)	
C5	0.76921 (19)	0.42384 (13)	0.03763 (9)	0.0193 (3)	
C6	0.8072 (2)	0.32072 (13)	0.07019 (9)	0.0210 (3)	
C7	0.8197 (2)	0.32839 (14)	0.16601 (9)	0.0240 (3)	
H7	0.9469	0.3811	0.1961	0.029*	
C8	0.6793 (2)	0.38156 (13)	0.19367 (9)	0.0226 (3)	
H8	0.5555	0.3313	0.1589	0.027*	
C9	0.7390 (2)	0.51454 (13)	0.09333 (9)	0.0203 (3)	
C10	0.7732 (2)	0.84986 (14)	−0.00181 (10)	0.0256 (3)	
C11	0.6960 (3)	0.93267 (16)	−0.04461 (13)	0.0430 (5)	
H11A	0.7555	1.0174	−0.0139	0.064*	
H11B	0.5628	0.9064	−0.0454	0.064*	
H11C	0.7187	0.9288	−0.1027	0.064*	
C12	0.6877 (2)	0.24719 (15)	−0.13283 (10)	0.0281 (3)	
C13	0.7670 (3)	0.17287 (17)	−0.18755 (11)	0.0388 (4)	
H13A	0.8635	0.1541	−0.1526	0.058*	
H13B	0.8201	0.2199	−0.2299	0.058*	
H13C	0.6693	0.0959	−0.2161	0.058*	
C14	0.9157 (3)	0.16750 (17)	0.20681 (10)	0.0327 (4)	
C15	0.8447 (3)	0.04084 (18)	0.22644 (12)	0.0477 (5)	
H15A	0.9488	0.0144	0.2444	0.072*	
H15B	0.7653	−0.0158	0.1759	0.072*	
H15C	0.7735	0.0405	0.2719	0.072*	
C16	0.6647 (2)	0.38600 (14)	0.28534 (9)	0.0216 (3)	
C17	0.7826 (2)	0.48387 (14)	0.34725 (9)	0.0227 (3)	
H17	0.8803	0.5474	0.3329	0.027*	
C18	0.7543 (2)	0.48648 (14)	0.42975 (9)	0.0226 (3)	
C19	0.6149 (2)	0.39279 (14)	0.45148 (9)	0.0221 (3)	
C20	0.5068 (2)	0.29293 (14)	0.39022 (10)	0.0231 (3)	
C21	0.5276 (2)	0.29052 (14)	0.30684 (9)	0.0235 (3)	
H21	0.4484	0.2239	0.2645	0.028*	
C22	0.9069 (2)	0.69928 (14)	0.49122 (10)	0.0253 (3)	
C23	1.0292 (2)	0.77456 (15)	0.57159 (10)	0.0307 (4)	
H23A	1.0586	0.8623	0.5690	0.046*	
H23B	1.1436	0.7560	0.5794	0.046*	
H23C	0.9659	0.7549	0.6192	0.046*	
C24	0.6593 (2)	0.36239 (15)	0.59285 (10)	0.0282 (3)	
C25	0.6109 (3)	0.3970 (2)	0.67600 (11)	0.0462 (5)	
H25A	0.4802	0.3516	0.6751	0.069*	
H25B	0.6337	0.4857	0.6866	0.069*	
H25C	0.6870	0.3765	0.7209	0.069*	
C26	0.3857 (2)	0.08583 (15)	0.40763 (11)	0.0316 (4)	
C27	0.2336 (3)	−0.00180 (19)	0.43981 (16)	0.0515 (5)	
H27A	0.1167	−0.0205	0.4009	0.077*	
H27B	0.2249	0.0355	0.4959	0.077*	
H27C	0.2592	−0.0780	0.4438	0.077*	

### Atomic displacement parameters (Å^2^)

**Table:**

	*U*^11^	*U*^22^	*U*^33^	*U*^12^	*U*^13^	*U*^23^
O1	0.0379 (6)	0.0226 (5)	0.0161 (5)	0.0125 (5)	0.0074 (4)	0.0032 (4)
O2	0.0397 (7)	0.0246 (6)	0.0242 (6)	0.0152 (5)	0.0085 (5)	0.0015 (4)
O3	0.0302 (6)	0.0191 (5)	0.0229 (5)	0.0072 (5)	−0.0005 (4)	0.0036 (4)
O4	0.0340 (7)	0.0263 (6)	0.0326 (7)	0.0069 (5)	−0.0023 (5)	−0.0008 (5)
O5	0.0308 (6)	0.0244 (5)	0.0187 (5)	0.0108 (5)	0.0084 (4)	0.0007 (4)
O6	0.0308 (7)	0.0324 (7)	0.0467 (8)	0.0069 (5)	0.0025 (6)	−0.0089 (5)
O7	0.0357 (6)	0.0257 (6)	0.0263 (6)	0.0140 (5)	0.0094 (5)	0.0092 (4)
O8	0.0437 (9)	0.0627 (9)	0.0511 (9)	0.0285 (8)	0.0027 (7)	0.0131 (7)
O9	0.0353 (6)	0.0261 (6)	0.0176 (5)	0.0098 (5)	−0.0010 (4)	−0.0006 (4)
O10	0.0376 (7)	0.0310 (6)	0.0267 (6)	0.0121 (5)	0.0029 (5)	0.0047 (5)
O11	0.0372 (6)	0.0392 (6)	0.0167 (5)	0.0239 (5)	0.0100 (5)	0.0093 (4)
O12	0.0768 (11)	0.0851 (11)	0.0371 (7)	0.0652 (9)	0.0248 (7)	0.0286 (7)
O13	0.0290 (6)	0.0289 (6)	0.0343 (6)	0.0101 (5)	0.0145 (5)	0.0107 (5)
O14	0.0421 (8)	0.0320 (7)	0.0747 (10)	0.0152 (6)	0.0248 (7)	0.0116 (6)
C1	0.0232 (8)	0.0208 (7)	0.0206 (7)	0.0068 (6)	0.0041 (6)	0.0002 (6)
C2	0.0179 (7)	0.0176 (7)	0.0237 (7)	0.0033 (6)	0.0021 (6)	0.0041 (6)
C3	0.0203 (7)	0.0243 (7)	0.0167 (7)	0.0041 (6)	0.0035 (6)	0.0036 (6)
C4	0.0181 (7)	0.0198 (7)	0.0192 (7)	0.0038 (6)	0.0043 (5)	−0.0008 (5)
C5	0.0177 (7)	0.0198 (7)	0.0187 (7)	0.0044 (6)	0.0041 (5)	0.0021 (5)
C6	0.0191 (7)	0.0216 (7)	0.0212 (7)	0.0053 (6)	0.0049 (6)	0.0025 (6)
C7	0.0274 (8)	0.0230 (7)	0.0227 (8)	0.0096 (6)	0.0056 (6)	0.0049 (6)
C8	0.0266 (8)	0.0217 (7)	0.0180 (7)	0.0070 (6)	0.0042 (6)	0.0021 (6)
C9	0.0199 (7)	0.0215 (7)	0.0171 (7)	0.0049 (6)	0.0028 (5)	0.0015 (5)
C10	0.0258 (8)	0.0209 (7)	0.0280 (8)	0.0051 (6)	0.0059 (7)	0.0036 (6)
C11	0.0446 (11)	0.0235 (9)	0.0547 (12)	0.0092 (8)	−0.0057 (9)	0.0075 (8)
C12	0.0339 (9)	0.0277 (8)	0.0218 (8)	0.0137 (7)	−0.0016 (7)	0.0001 (6)
C13	0.0530 (12)	0.0361 (10)	0.0305 (9)	0.0241 (9)	0.0042 (8)	−0.0031 (7)
C14	0.0432 (11)	0.0413 (10)	0.0187 (8)	0.0229 (9)	0.0040 (7)	0.0033 (7)
C15	0.0823 (16)	0.0368 (10)	0.0304 (10)	0.0338 (11)	0.0007 (10)	0.0037 (8)
C16	0.0247 (8)	0.0247 (7)	0.0170 (7)	0.0109 (6)	0.0039 (6)	0.0032 (6)
C17	0.0236 (8)	0.0238 (7)	0.0210 (7)	0.0083 (6)	0.0044 (6)	0.0039 (6)
C18	0.0253 (8)	0.0247 (7)	0.0179 (7)	0.0113 (6)	0.0007 (6)	0.0006 (6)
C19	0.0284 (8)	0.0284 (8)	0.0166 (7)	0.0172 (7)	0.0068 (6)	0.0060 (6)
C20	0.0216 (7)	0.0255 (8)	0.0261 (8)	0.0110 (6)	0.0078 (6)	0.0075 (6)
C21	0.0245 (8)	0.0243 (7)	0.0204 (7)	0.0083 (6)	0.0025 (6)	0.0013 (6)
C22	0.0247 (8)	0.0284 (8)	0.0228 (8)	0.0089 (7)	0.0083 (6)	0.0015 (6)
C23	0.0298 (9)	0.0314 (9)	0.0264 (8)	0.0081 (7)	0.0035 (7)	−0.0023 (7)
C24	0.0311 (9)	0.0351 (9)	0.0240 (8)	0.0166 (7)	0.0064 (7)	0.0106 (7)
C25	0.0596 (13)	0.0746 (14)	0.0213 (9)	0.0421 (12)	0.0116 (8)	0.0148 (9)
C26	0.0300 (9)	0.0291 (9)	0.0346 (9)	0.0084 (7)	0.0059 (7)	0.0073 (7)
C27	0.0491 (12)	0.0397 (11)	0.0755 (15)	0.0144 (10)	0.0306 (11)	0.0288 (10)

### Geometric parameters (Å, °)

**Table:**

O1—C9	1.3704 (17)	C8—H8	1.0000
O1—C8	1.4322 (18)	C10—C11	1.486 (2)
O2—C6	1.2143 (17)	C11—H11A	0.9800
O3—C10	1.3773 (18)	C11—H11B	0.9800
O3—C2	1.3830 (17)	C11—H11C	0.9800
O4—C10	1.1911 (19)	C12—C13	1.494 (2)
O5—C12	1.3709 (19)	C13—H13A	0.9800
O5—C4	1.3916 (16)	C13—H13B	0.9800
O6—C12	1.196 (2)	C13—H13C	0.9800
O7—C14	1.351 (2)	C14—C15	1.488 (3)
O7—C7	1.4265 (18)	C15—H15A	0.9800
O8—C14	1.197 (2)	C15—H15B	0.9800
O9—C22	1.3743 (19)	C15—H15C	0.9800
O9—C18	1.3867 (18)	C16—C21	1.385 (2)
O10—C22	1.1910 (19)	C16—C17	1.397 (2)
O11—C24	1.3525 (19)	C17—C18	1.382 (2)
O11—C19	1.3886 (17)	C17—H17	0.9500
O12—C24	1.185 (2)	C18—C19	1.385 (2)
O13—C26	1.371 (2)	C19—C20	1.383 (2)
O13—C20	1.3941 (18)	C20—C21	1.380 (2)
O14—C26	1.191 (2)	C21—H21	0.9500
C1—C2	1.377 (2)	C22—C23	1.492 (2)
C1—C9	1.383 (2)	C23—H23A	0.9800
C1—H1	0.9500	C23—H23B	0.9800
C2—C3	1.394 (2)	C23—H23C	0.9800
C3—C4	1.370 (2)	C24—C25	1.492 (2)
C3—H3	0.9500	C25—H25A	0.9800
C4—C5	1.410 (2)	C25—H25B	0.9800
C5—C9	1.4072 (19)	C25—H25C	0.9800
C5—C6	1.469 (2)	C26—C27	1.491 (2)
C6—C7	1.533 (2)	C27—H27A	0.9800
C7—C8	1.514 (2)	C27—H27B	0.9800
C7—H7	1.0000	C27—H27C	0.9800
C8—C16	1.5013 (19)		
			
C9—O1—C8	115.21 (11)	H13A—C13—H13C	109.5
C10—O3—C2	120.74 (12)	H13B—C13—H13C	109.5
C12—O5—C4	118.54 (12)	O8—C14—O7	122.91 (17)
C14—O7—C7	116.89 (13)	O8—C14—C15	127.04 (17)
C22—O9—C18	120.25 (12)	O7—C14—C15	110.04 (17)
C24—O11—C19	118.70 (12)	C14—C15—H15A	109.5
C26—O13—C20	116.82 (12)	C14—C15—H15B	109.5
C2—C1—C9	117.87 (13)	H15A—C15—H15B	109.5
C2—C1—H1	121.1	C14—C15—H15C	109.5
C9—C1—H1	121.1	H15A—C15—H15C	109.5
C1—C2—O3	122.15 (13)	H15B—C15—H15C	109.5
C1—C2—C3	122.11 (13)	C21—C16—C17	120.62 (13)
O3—C2—C3	115.47 (13)	C21—C16—C8	117.82 (13)
C4—C3—C2	118.70 (13)	C17—C16—C8	121.54 (14)
C4—C3—H3	120.6	C18—C17—C16	118.70 (14)
C2—C3—H3	120.6	C18—C17—H17	120.7
C3—C4—O5	116.72 (13)	C16—C17—H17	120.7
C3—C4—C5	122.16 (13)	C17—C18—C19	121.01 (14)
O5—C4—C5	120.86 (13)	C17—C18—O9	123.72 (14)
C9—C5—C4	116.26 (13)	C19—C18—O9	115.19 (13)
C9—C5—C6	119.47 (13)	C20—C19—C18	119.42 (13)
C4—C5—C6	124.15 (12)	C20—C19—O11	120.74 (14)
O2—C6—C5	125.38 (13)	C18—C19—O11	119.56 (13)
O2—C6—C7	120.45 (13)	C21—C20—C19	120.55 (14)
C5—C6—C7	114.16 (12)	C21—C20—O13	121.56 (14)
O7—C7—C8	107.60 (12)	C19—C20—O13	117.75 (13)
O7—C7—C6	109.39 (12)	C20—C21—C16	119.51 (14)
C8—C7—C6	108.99 (12)	C20—C21—H21	120.2
O7—C7—H7	110.3	C16—C21—H21	120.2
C8—C7—H7	110.3	O10—C22—O9	123.93 (14)
C6—C7—H7	110.3	O10—C22—C23	127.63 (15)
O1—C8—C16	108.45 (11)	O9—C22—C23	108.43 (13)
O1—C8—C7	107.80 (12)	C22—C23—H23A	109.5
C16—C8—C7	115.37 (12)	C22—C23—H23B	109.5
O1—C8—H8	108.3	H23A—C23—H23B	109.5
C16—C8—H8	108.3	C22—C23—H23C	109.5
C7—C8—H8	108.3	H23A—C23—H23C	109.5
O1—C9—C1	115.19 (12)	H23B—C23—H23C	109.5
O1—C9—C5	121.95 (13)	O12—C24—O11	122.55 (15)
C1—C9—C5	122.86 (13)	O12—C24—C25	127.56 (16)
O4—C10—O3	123.10 (14)	O11—C24—C25	109.88 (14)
O4—C10—C11	127.33 (15)	C24—C25—H25A	109.5
O3—C10—C11	109.57 (13)	C24—C25—H25B	109.5
C10—C11—H11A	109.5	H25A—C25—H25B	109.5
C10—C11—H11B	109.5	C24—C25—H25C	109.5
H11A—C11—H11B	109.5	H25A—C25—H25C	109.5
C10—C11—H11C	109.5	H25B—C25—H25C	109.5
H11A—C11—H11C	109.5	O14—C26—O13	122.89 (16)
H11B—C11—H11C	109.5	O14—C26—C27	126.64 (17)
O6—C12—O5	122.57 (14)	O13—C26—C27	110.47 (15)
O6—C12—C13	127.38 (16)	C26—C27—H27A	109.5
O5—C12—C13	110.04 (15)	C26—C27—H27B	109.5
C12—C13—H13A	109.5	H27A—C27—H27B	109.5
C12—C13—H13B	109.5	C26—C27—H27C	109.5
H13A—C13—H13B	109.5	H27A—C27—H27C	109.5
C12—C13—H13C	109.5	H27B—C27—H27C	109.5

### Hydrogen-bond geometry (Å, °)

**Table:**

Cg3 is the centroid of the C16–C21 ring.

**Table:**

*D*—H···*A*	*D*—H	H···*A*	*D*···*A*	*D*—H···*A*
C1—H1···O6^i^	0.95	2.53	3.2847 (19)	136
C8—H8···O3^i^	1.00	2.33	3.2833 (18)	158
C11—H11A···O2^ii^	0.98	2.40	3.347 (2)	161
C13—H13A···O4^iii^	0.98	2.44	3.417 (2)	176
C15—H15B···O4^iv^	0.98	2.59	3.232 (2)	124
C23—H23B···Cg3^v^	0.98	2.86	3.7598 (18)	153

Symmetry codes: (i) −*x*+1, −*y*+1, −*z*; (ii) *x*, *y*+1, *z*; (iii) −*x*+2, −*y*+1, −*z*; (iv) *x*, *y*−1, *z*; (v) −*x*+2, −*y*+1, −*z*+1.
